# Confocal Imaging: Blood and Lymphatic Capillaries

**DOI:** 10.1100/tsw.2006.12

**Published:** 2006-01-17

**Authors:** Yingjie Cui

**Affiliations:** Department of Thoracic Surgery, First Hospital of Beijing University, Beijing, China

**Keywords:** confocal microscopy, blood vessel, lymphatic vessel, lyve-1, prox-1, podoplanin, vascular endothelial growth factor receptor-2, vascular endothelial growth factor receptor-3, Von Willebrand factor, platelet endothelial cell adhesion molecule

## Abstract

Traditional imaging techniques are quite limited for the study of the relationship between blood vessels and lymphatic vessels. Therefore, a new imaging technique is required based on blood vessel and lymphatic endothelial-specific molecular markers. In this short report, vascular molecular markers are reviewed and a new molecular imaging technique for blood vessel and lymphatic co-staining is introduced.

## INTRODUCTION

Although we have known of the existence of lymphatic vessels for over 300 years[1], the relationship between blood vessels and lymphatic vessels was largely unknown. For a long time, our knowledge was restricted to the fact that two specialized vascular systems have evolved for effective circulation: the blood vasculature, which delivers oxygen and nutrients and carries away waste products for detoxification and replenishment; and the lymphatic vasculature, which returns the protein-rich extruded fluid to the bloodstream[2]. Lymphatic dysfunction is known as a cause of edema, but was never linked to blood vessel injury until recent studies in cardiopulmonary pathophysiology showed that perivascular lymphatic dysfunction may cause blood vessel injury in heart and lung[3,4,5]. Unfortunately, the mechanism of blood vessel injury by lymphatic dysfunction is unknown, in part, due to poor technology in identifying lymphatic endothelium from blood endothelium.

Recent advances in lymphatic biology and imaging technology have brought great progress in vascular study, particularly in the mechanism of lymphangiogenesis. In addition, the study of the mechanism of blood vessel injury associated with lymphatic dysfunction is becoming possible. This report will introduce recent progress in blood and lymphatic endothelial cell molecular markers and imaging techniques.

## MATERIALS AND METHODS

Mouse tail skin has been widely used in the study of lymphatic biology. Therefore, lymphatic and blood vessel costaining in the mouse tail skin model was demonstrated in this study. Balb/c mouse was anesthetized by intraperitoneal injection of ketamine and xylozine; 0.5~1 cm mouse tail was sharp cut, and was put in frozen section medium, followed by sectioning in 60 μm. After fixing with cold acetone for 5 min and blocking in 10% goat serum for 90 min, the section was incubated with primary antibodies of rabbit antimouse LYVE-1 antibody and rat antimouse CD31 antibody at 4°C for overnight. After washing in PBS, the section was incubated in the secondary antibodies of goat antirabbit IgG (green) and goat antirat IgG (red), for 1 h at room temperature. Eventually, the section was preserved with mounting medium without DAPI, and coverslip was sealed by nail polish. The image below was taken with confocal laser scanning microscope (amplification ×200).

## RESULTS

As shown in the figure, green color represents lymphatic vessels and red color represents blood vessels.

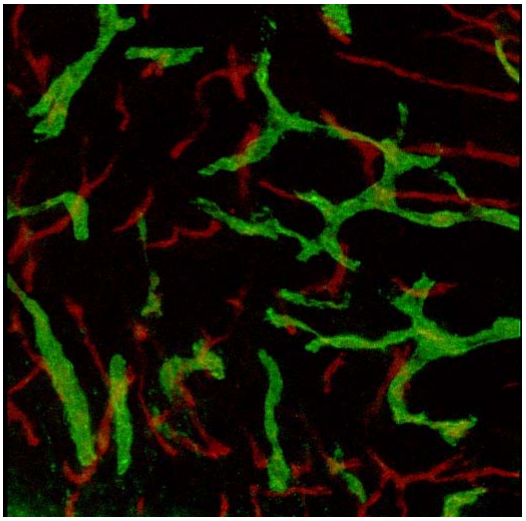

## DISCUSSION

There are various blood and lymphatic endothelial molecular markers for different utilities. For example, specific lymphatic endothelium markers include lymphatic vessel endothelial receptor-1 (LYVE-1), prospero-related homeobox gene-1 (Prox-1), podoplanin, vascular endothelial growth factor receptor-3 (VEGFR-3), and others.

LYVE-1 receptor is a type I integral membrane polypeptide expressed on the cell surface, which is abundant in spleen, lymph node, heart, lung, and fetal liver; less abundant in appendix, bone marrow, placenta, muscle, and adult liver; and absent in peripheral blood lymphocytes, thymus, brain, kidney, and pancreas. Expression of LYVE-1 is largely restricted to endothelial cells lining lymphatic vessels and splenic sinusoidal endothelial cells. The highest concentration of LYVE-1 expression was found in submucosal lymph vessels underlying smooth muscle in the colon, and the lacteal vessels of intestinal villi that transport dietary lipid absorbed from the small intestine[6].

Prox-1 is a marker for the subpopulation of endothelial cells that bud and sprout to give rise to the lymphatic system during early development. Targeted deletion of the prox-1 gene does not affect development of the blood vascular system, but the budding and sprouting of the developing lymphatics is ablated, suggesting that prox-1 plays an important role in lymphatic system development[7].

Podoplanin is a surface glycoprotein that was recently cloned as a cell surface protein expressed on normal rat kidney podocytes. Podoplanin is expressed on epithelial cells of the choroide plexus and lymphatic endothelial cells, but the lung is a major site of its expression. Microscopic immunohistology demonstrates the specificity of podoplanin expression on lymphatic, but not blood vasculature, endothelia in the skin. Also, podoplanin was found to be expressed on PAL-E-negative vessels and to colocalize with VEGFR-3[8].

VEGFR-3 is mainly on lymphatic vessels, but also reactivated in blood vessels in pathological conditions[9]. There are other less-specific markers that have been used in the past to distinguish between the blood vascular and lymphatic endothelia, including antibodies against basement membrane components such as collagen type IV, fibronectin, vitronectin and laminin, and desmoplakin[10]. In our experience, LYVE-1 is the best choice for lymphatic staining because of its strong signal and specificity.

Blood vessel endothelium markers include vascular endothelial growth factor receptor-2 (VEGFR-2), Von Willebrand factor (factor VIII), VE-cadherin, platelet endothelial cell adhesion molecule (PECAM / CD31), CD34, and others.

VEGFR-2 is mainly expressed in blood vessel endothelium, although VEGFR-2 is also expressed in lymphatic vessels and even plays an important role in lymphangiogenesis[11]. The result of blood vessel–specific staining with anti-VEGFR-2 antibody is similar to using PECAM/CD31 antibody.

Von Willebrand factor (vWF) is an important factor of the blood coagulation system. It facilitates adherence of blood platelets to the injured vessel wall. vWF antigen is synthesized by blood endothelial cells and is stored in the Weibel-Plalade bodies of these cells. Other cells in addition to endothelial cells also release vWF, for example, platelets and megakaryocytes[12].

VE-cadherin is an endothelial-specific cadherin that, in the embryo, is expressed at very early stages (E7.5) of vascular development in mesodermal cells of the yolk sac mesenchyme. At later embryonic stages, VE-cadherin expression is restricted to the peripheral layer of blood islands that gives rise to endothelial cells[13].

PECAM/CD31 belongs to the immunoglobulin superfamily and is a transmembrane protein organized in six Ig-like homology domains and promotes both homotypic adhesion, via another identical PECAM molecule, and heterotypic adhesion, via binding to glycosaminoglycans. PECAM is an endothelial cell adhesion molecule, which mainly expresses in blood vessels, although it is also expressed in lymphatic vessels. CD34 is a cell surface antigen of approximately 110 kDa and is expressed selectively on hematopoitic progenitor cells and vascular endothelial cells[14,15].

In the author's experience, selecting specific blood vessel and lymphatic markers depends on animal species and different tissue. For example, selecting CD31 and LYVE-1 is a good choice for blood vessel and lymphatic vessel costaining in mouse skin, but VEGFR-2 and LYVE-1 may be a better choice for blood vessel and lymphatic vessel costaining in mouse trachea.

## BIOSKETCH

**Yingjie Cui** is Senior Surgeon and Researcher, Department of Thoracic Surgery, First Hospital of Beijing University and Research Fellow, Division of Surgical Research, Feinstein Institute for Medical Research, North Shore-LIJ Health System. Past and current research interests include surgical complication, acute lung injury, sepsis, surgical wound healing, and lymphatic biology.
